# Genome Sequence of *Mycoplasma meleagridis* Type Strain 17529

**DOI:** 10.1128/genomeA.00484-15

**Published:** 2015-05-21

**Authors:** Elhem Yacoub, Pascal Sirand-Pugnet, Alain Blanchard, Boutheina Ben Abdelmoumen Mardassi

**Affiliations:** a Unit of Mycoplasmas, Laboratory of Molecular Microbiology, Vaccinology, and Biotechnology Development, Institut Pasteur de Tunis, Tunis, Tunisia; b INRA, UMR 1332 de Biologie du Fruit et Pathologie, Villenave d’Ornon, France; c Université de Bordeaux, UMR 1332 de Biologie du Fruit et Pathologie, Villenave d’Ornon, France

## Abstract

*Mycoplasma meleagridis* is a prominent turkey bacterial pathogen associated with airsacculitis and reproductive disorders. Notwithstanding the economic losses caused by *M. meleagridis*, its genome has still not been sequenced. For a better understanding of its genetic background and pathogenicity mechanisms, we sequenced the genome of *M. meleagridis* type strain ATCC 25294.

## GENOME ANNOUNCEMENT

*Mycoplasma meleagridis* is usually reported as a specific pathogen of turkeys that causes embryo mortality, skeletal abnormalities, and poor growth performance in the infected progeny (1). Nevertheless, its occasional isolation from chickens showing signs of respiratory disease and/or egg production losses suggests that *M. meleagridis* might also be a pathogen in chickens. This assumption was formally demonstrated in 2011 and opened many questions about transmission and adaptation to chickens as a new host (2). To start answering these questions, the availability of the complete genome sequence of *M. meleagridis* was required. Here, we determined the complete genome sequence of *M. meleagridis* type strain ATCC 25294, originally isolated from infected turkeys.

The genome sequencing of *M. meleagridis* ATCC 25294 strain was achieved at the Genome-Transcriptome facility of Bordeaux (http://www.pgtb.u-bordeaux2.fr) using the Illumina MiSeq version 2 sequencer and by combining mate-pair and paired-end libraries. *De novo* assembly was performed at the Bordeaux Bioinformatics Center (http://www.cbib.u-bordeaux2.fr/) using CLC and ABySS softwares. Coding sequences (CDSs) were predicted with the fully automated Rapid Annotations using Subsystems Technology (RAST) server (3). This initial annotation was improved by manual curation. The RNAmmer 2.1 software (4) was run to verify rRNAs, and the Aragorn software (5) was used to predict tRNAs and transfer-messenger RNAs (tmRNAs). The annotated genome was integrated into the MolliGen database (http://cbi.labri.fr/outils/molligen/), which is dedicated to the comparative genomics of *Mollicutes* genomes (6). Analysis of the *M. meleagridis* genome was supported by tools available in RAST/SEED and MolliGen.

*De novo* assembly of the *M. meleagridis* genome resulted in 22 contigs organized in 7 scaffolds. The genome sequence is composed of 634,182 bp, with an overall G+C content of 26.02%. It contains 505 predicted CDSs, representing a coding density of 91.49%. The *M. meleagridis* genome includes one copy each of the 16S and 23S rRNA genes and two copies of the 5S rRNA gene. A set of 33 tRNA genes, corresponding to all amino acids, was also characterized.

The origin of replication of *M. meleagridis* has not been experimentally identified. However, as described for other bacterial genomes, the tandem arrangement of the *dnaA* (MMELEA_00800) and *dnaN* (MMELEA_00810) genes was found (7, 8).

A phylogenetic tree inferred from the 16S rRNA gene sequences of multiple mycoplasma species showed that *M. meleagridis* belongs to the Hominis group of mycoplasmas. Analysis of the *M. meleagridis* genome revealed that the arginine dihydrolase pathway is complete. Indeed, the genes *arcA*, *arcB*, and *arcC* encoding arginine deiminase (MMELEA_04580), ornithine carbamoyltransferase (MMELEA_04570), and carbamate kinase (MMELEA_04560), respectively, were identified. This finding is in accordance with the ability of *M. meleagridis* to alkalinize culture medium containing arginine (9).

This is the first genome sequence of the *M. meleagridis* species, which will be helpful for a better understanding of the genetic basis of the virulence of the pathogen. Further genome sequencing of *M. meleagridis* strains isolated from chickens will be necessary to address the question of host specificity.

### Nucleotide sequence accession numbers.

This whole-genome shotgun project has been deposited at DDBJ/EMBL/GenBank under the accession no. JZXN00000000. The version described in this paper is version JZXN01000000.
